# Pain and Patient‐Reported Physical Function Did Not Differ Among Body Composition Profiles in Patients With Hip Osteoarthritis

**DOI:** 10.1002/acr.80058

**Published:** 2026-05-05

**Authors:** Alexandra Ryan, Boliang Wang, Fiona Dobson, Ben Kirk, Travis Haber, Kim L. Bennell, Shirley P. Yu, Kim Allison, Ricardo J. S. Costa, Gabrielle Knox, Michelle Hall

**Affiliations:** ^1^ Centre for Health Exercise and Sports Medicine, Department of Physiotherapy University of Melbourne Melbourne Victoria Australia; ^2^ Discipline of Exercise and Sport Science, School of Health Sciences, Faculty of Medicine and Health The University of Sydney Camperdown New South Wales Australia; ^3^ Musculoskeletal Research Hub, Charles Perkins Centre, The University of Sydney Sydney New South Wales Australia; ^4^ Department of Medicine, Western Health, Melbourne Medical School University of Melbourne Melbourne Victoria Australia; ^5^ Australian Institute for Musculoskeletal Science (AIMSS) University of Melbourne and Western Health Melbourne Victoria Australia; ^6^ Sydney Musculoskeletal Health, Kolling Institute, Faculty of Medicine and Health The University of Sydney Sydney New South Wales Australia; ^7^ BASE Facility, Department of Nutrition Dietetics & Food Monash University Melbourne Victoria Australia

## Abstract

**Objective:**

To explore whether higher body fat and lower lean mass are associated with greater pain and worse patient‐reported physical function in individuals with hip osteoarthritis (OA). A secondary aim was to examine whether pain and patient‐reported physical function differ according to four body composition profiles: high body fat, low lean mass, both, or neither.

**Methods:**

This cross‐sectional study analyzed baseline data from two randomized controlled trials involving 160 individuals with symptomatic hip OA (mean ± SD age 61.4 ± 6.2 years; 65% were female). Body fat mass percentage and appendicular lean mass (ALM) were assessed using dual‐energy x‐ray absorptiometry. Participants were categorized into four body composition profiles: high body fat (n = 86), low lean mass (n = 11), combined (n = 8), or neither (n = 55). Pain and patient‐reported physical function were assessed using the Numeric Rating Scale and the Western Ontario and McMaster Universities OA Index, respectively. Associations between body composition measures and symptom outcomes were examined using linear regression, and differences across body composition profiles were analyzed using one‐way analysis of variance.

**Results:**

Neither body fat mass percentage nor ALM was significantly associated with pain or patient‐reported physical function. No significant differences in pain or patient‐reported physical function were observed across body composition profiles.

**Conclusion:**

Body composition was not associated with pain or patient‐reported physical function in this cross‐sectional sample of individuals with hip OA. Future research could incorporate more precise measures of muscle mass and explore functional phenotypes, including those based on muscle strength, to better understand symptom burden.

## INTRODUCTION

Hip osteoarthritis (OA) is a leading cause of pain and disability worldwide,^1^ with limited effective nonsurgical treatment options.^2^ Although there is evidence that altered body composition characteristics, such as high fat mass or low muscle mass, may influence OA progression,^3^, ^4^ less is known about how specific body composition measures relate to symptoms such as pain and physical function limitations,^5^, ^6^ particularly in hip OA. Understanding these cross‐sectional associations may help guide future research aimed at optimizing management strategies, such as targeted weight loss or exercise interventions, and identifying individuals most likely to benefit from such approaches.

**SIGNIFICANCE & INNOVATIONS**

- To our knowledge, this study is the first to examine associations between dual‐energy x‐ray absorptiometry–derived body composition measures and pain and patient‐reported physical function in individuals with clinically diagnosed hip osteoarthritis.
- Body fat mass percentage and appendicular lean mass were not associated with pain severity or worse patient‐reported physical function in hip osteoarthritis, suggesting that body composition alone may have limited relevance for these symptom outcomes in this population.
- Pain severity and patient‐reported physical function did not differ across body composition profiles defined by high body fat, low lean mass, their combination, or neither, questioning the clinical utility of body composition–based phenotyping for symptom stratification in hip osteoarthritis.
- By demonstrating limited associations between body composition and symptom burden, this study highlights the need for future research to examine other muscle‐related characteristics, such as muscle strength, physical performance, and muscle quality, in hip osteoarthritis.

Excess adiposity has traditionally been viewed as a mechanical stressor on weight‐bearing joints,^7^ but adipose tissue also secretes pro‐inflammatory mediators that may increase nociceptive sensitivity.^8^, ^9^ Through both increased joint loading and obesity‐related metabolic inflammation (eg, adipokine and cytokine signaling), adiposity may contribute to pain sensitization and functional limitation in OA.^10^ In knee OA, body fat mass percentage has been found to be significantly associated with pain intensity,^11^ and higher fat mass is associated with lower physical function.^5^ However, whether similar relationships exist in hip OA remains unclear. This is particularly relevant because obesity appears to be more strongly associated with the development of knee OA than hip OA,^12^ suggesting that the role of adiposity may differ between joints. Moreover, evidence supporting weight loss interventions for symptom management in hip OA is comparatively limited and less consistent than in knee OA.^13^, ^14^

Muscle mass is another key component of body composition, as skeletal muscle contributes to the joint stability and force generation required for movement. Greater lower‐limb muscle mass has been inversely associated with pain severity in individuals with knee OA.^15^, ^16^ However, direct evidence examining muscle mass in relation to pain and physical function in hip OA is limited. Although some studies have examined muscle volume, which is related but not equivalent to muscle mass,^17^ findings suggest that hip muscle volume may not be substantially reduced in hip OA populations.^18^ In contrast, a cross‐sectional study indicates that greater hip adductor volume may be associated with better function in individuals with hip OA.^19^ These uncertainties regarding the role of muscle in hip OA highlight the need for research assessing limb muscle mass to clarify its relationship with pain and physical function.

Sarcopenia is characterized by declines in skeletal muscle mass, strength, and physical function,^20^ whereas sarcopenic obesity refers to the coexistence of sarcopenia and obesity. Definitions vary across studies and may rely on body mass index (BMI) or body composition measures (eg, body fat mass percentage).^21^, ^22^ In knee OA, sarcopenia, obesity, and sarcopenic obesity have been commonly studied and associated with pain and physical dysfunction,^23^, ^24^ with some evidence suggesting that sarcopenic obesity may confer a greater symptom burden than either condition alone or neither.^23^, ^24^, ^25^ However, the existing evidence is largely limited to knee OA or mixed samples of hip and knee OA.^24^ Interpretation is further complicated by variability in the definitions of sarcopenia and sarcopenic obesity. A recent systematic review found that only one‐third of OA studies applied a full diagnostic definition for sarcopenia or sarcopenic obesity,^26^ and the remaining studies defined sarcopenia solely based on body composition measures (ie, lean mass).^26^ This likely reflects practical limitations, as existing datasets often lack both strength and physical function assessments. Consequently, the reported prevalence of sarcopenia (16.7%) and sarcopenic obesity (14.0%) in OA may overestimate the true proportion when full contemporary diagnostic criteria are applied.^23^, ^24^, ^26^ Nevertheless, focusing on individual body composition components such as lean mass and body fat mass percentage remains valuable, as these measures reflect distinct biologic characteristics of muscle and adiposity that may be associated with pain and physical function in OA.^5^, ^6^

In our previous two hip OA studies,^14^, ^27^ baseline body composition was assessed using dual‐energy x‐ray absorptiometry (DEXA). Because of the absence of muscle strength and physical performance data, we do not classify sarcopenia in the current study. Instead, we use appendicular lean mass (ALM), a surrogate for muscle mass assessed by DEXA,^20^, ^22^ along with body fat mass percentage to investigate associations between body composition and hip OA symptoms. Our study aims to (1) examine the associations of ALM and body fat mass percentage with symptom severity and (2) determine whether pain severity is greater and patient‐reported physical function is worse in individuals with (i) high body fat, (ii) low lean mass, or (iii) both compared with those with neither and whether the combination of high body fat and low lean mass is associated with more severe symptoms than either characteristic alone. Clarifying these relationships is clinically relevant because differences in body composition may contribute to heterogeneity in symptom presentation among people with hip OA and may help clinicians better stratify patients and tailor nonsurgical management strategies.

## MATERIALS AND METHODS

This cross‐sectional study used baseline measurements collected before intervention allocation from a subsample of 160 participants who underwent DEXA scans in two randomized controlled trials: 95 participants from a weight loss trial (total N = 101)^14^ and 65 participants from an exercise trial (total N = 196).^27^ DEXA data were unavailable for some participants because of COVID‐19 restrictions; therefore, the present analysis included only participants with available DEXA measurements (N = 160). Recruitment occurred via community advertisements from May 2021 to January 2023^14^ and from September 2019 to September 2022^27^ in Sydney and Melbourne, Australia. Eligibility criteria were (1) age ≥45 years, (2) hip pain history ≥3 months, (3) pain in the groin or hip region on most days of the past month, (4) average pain score ≥4 on an 11‐point scale (0 = no pain and 10 = worst pain imaginable) over the previous week, and (5) access to an internet‐connected device. Exclusion criteria included (1) previous hip replacement, (2) hip surgery in the past 6 months, (3) planned back/lower‐limb or bariatric surgery within 12 months, (4) self‐reported inflammatory arthritis, (5) neurologic conditions affecting the lower limbs, (6) current or planned pregnancy, and (7) inability to communicate in English. Ethical approval was obtained from the Human Research Ethics Committee of The University of Melbourne (IDs: 20516 and 12710).^14^, ^27^

Body composition was assessed by DEXA using GE Lunar iDXA narrow‐angle densitometers at one clinic in Melbourne, and Hologic densitometers running Apex software versions 5.6.0.7 and 5.6.0.5 at two clinics in Sydney. All measurements and analyses were conducted according to manufacturer guidelines and standard protocols to minimize interdevice variability. Lean mass and body fat mass percentage were derived from the scans, and ALM was calculated as the sum of lean mass in the upper and lower limbs.^22^ Participants were categorized into four body composition groups: (1) high body fat alone, defined as BMI ≥30 kg/m^2^ with body fat mass percentage ≥35% in female patients or ≥25% in male patients^22^; (2) low lean mass alone, defined as an ALM index (ALM/BMI) < 0.512 in female patients and <0.789 in male patients^22^; (3) combined, meeting both criteria; and (4) neither high body fat nor low lean mass. These body composition indices are based on guidelines from the Foundation for the National Institutes of Health for defining sarcopenia, obesity, and sarcopenic obesity,^22^ although the current study does not include objective measures of muscle strength or performance‐based physical function (eg, chair‐stand or gait speed), which are often used alongside ALM indices in sarcopenia definitions. The ALM/BMI index was used to account for differences in body size when categorizing low lean mass.

Pain was measured using an 11‐point Numeric Rating Scale (0 = no pain and 10 = worst pain imaginable),^28^ with participants reporting their average overall hip pain intensity over the previous week. Patient‐reported physical function was assessed using the Western Ontario and McMaster Universities OA Index (WOMAC) physical function subscale (0–68, higher scores indicate worse function), which assesses difficulty performing daily activities over the past 48 hours.^29^ Both questionnaires were self‐administered by participants as part of the baseline assessment and completed electronically via an online survey platform (REDCap).

Data were analyzed using IBM SPSS Statistics version 29.0.1.0. Statistical significance was set at *P* < 0.05. Analyses were conducted using complete‐case analysis, including only participants with complete data for the variables included in each model; therefore, no imputation procedures were applied.

Linear regression models were used to examine the associations between body fat mass percentage and ALM (independent variables) and clinical outcomes (pain and patient‐reported physical function; dependent variables). For regression analyses, raw ALM was used instead of ALM/BMI to allow clinically interpretable regression coefficients. Both unadjusted models and models adjusted for age, study site, symptom duration, and Kellgren‐Lawrence (KL) grade were performed for the full cohort, and further analyses were stratified by sex to explore potential sex differences. As an additional sensitivity analysis, regression models were repeated using lower‐limb lean mass (ie, sum of lean mass from both legs) and upper‐limb lean mass (ie, sum of lean mass from both arms) separately to explore whether region‐specific lean mass influenced the results. Residuals from each model were inspected and met the assumptions of linear regression.^30^

One‐way analysis of variance was used to compare average pain and patient‐reported physical function across the four body composition groups. Prespecified pairwise comparisons were performed using the group with neither high body fat nor low lean mass as the reference, and mean differences with 95% confidence intervals (CIs) were estimated for the high body fat alone, low lean mass alone, and combined groups. Additional prespecified comparisons were conducted for the combined group relative to each of the other three groups.

## RESULTS

A total of 160 individuals with symptomatic hip OA were included. Participant characteristics are presented in Table 1.

**Table 1:** Participant characteristics*

Characteristics	Total (N = 160)
Age, mean (SD), y	61.4 (6.2)
Sex (female), n (%)	104 (65)
Height, mean (SD), m	1.7 (0.1)
Weight, mean (SD), kg	89.3 (16.6)
BMI, mean (SD), kg/m^2^	31.5 (5.1)
Unilateral symptoms, n (%)	99 (62)
Symptom duration, mean (SD), y	4.7 (5.8)
Right hip, n (%)	93 (58)
NRS pain (0–10), mean (SD)	5.6 (1.5)
WOMAC function (0–68), mean (SD)	22.1 (10.5)
KL grade, n (%) (n = 157)^a^	
Grade 0	2 (1)
Grade 1	14 (9)
Grade 2	84 (53)
Grade 3	42 (26)
Grade 4	15 (9)
Employment, n (%)	
Currently employed	97 (61)
Retired (not because of health reasons)	50 (31)
Unemployed/student	6 (4)
Homemaker	5 (3)
Unable to work because of health reasons	2 (1)
Education, n (%)	
<3 years of high school	2 (1)
≥3 years of high school	15 (9)
Some education beyond high school	55 (34)
Completed tertiary or higher education	88 (55)
DEXA acquisition site, n (%)	
Monash University, VIC	131 (82)
Castlereagh Imaging, NSW	18 (11)
Measure Up, NSW	11 (7)
Body fat mass percentage, mean (SD)	42.2 (8.1)
ALM/BMI, mean (SD)^b^	0.8 (0.2)

* ALM, appendicular lean mass; BMI, body mass index; DEXA, dual‐energy x‐ray absorptiometry; KL, Kellgren‐Lawrence; NRS, Numeric Rating Scale; NSW, New South Wales; VIC, Victoria; WOMAC, Western Ontario and McMaster Universities Osteoarthritis Index.

^a^ KL grade was unavailable for three participants because of unavailable radiographs during the COVID‐19 period.

^b^ Cutoff criteria for low ALM/BMI (female and male patients, respectively) was <0.512 and <0.789.

No statistically significant relationships were observed between ALM or body fat mass percentage and pain severity or patient‐reported physical function, as illustrated in Figure 1. These findings were consistent across sex‐stratified analyses in men and women, and additional adjustment for age, study site, symptom duration, and KL grade did not materially alter the observed associations (Table 2). Additional sensitivity analyses using lower‐ and upper‐limb lean mass separately yielded similar results (Supplementary Table 1).

**Figure 1:**
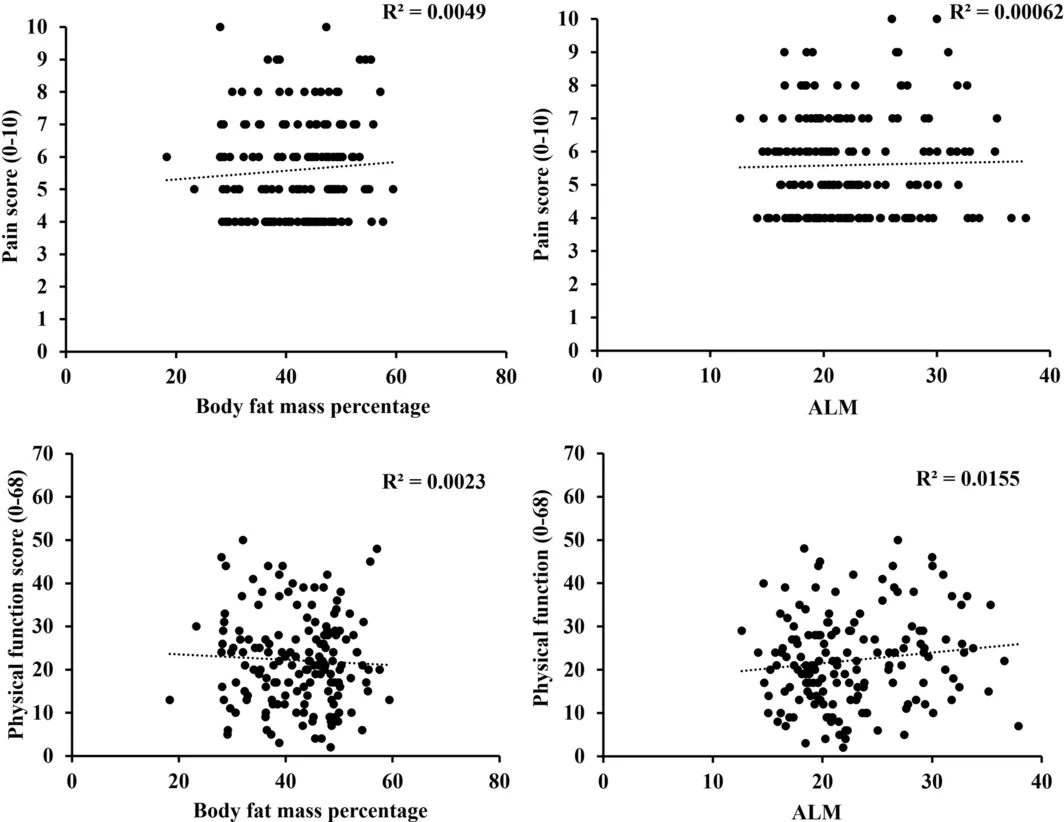
Unadjusted associations of body fat mass percentage and ALM with pain (Numeric Rating Scale, 0–10) and patient‐reported physical function (Western Ontario and McMaster Universities Osteoarthritis Index, 0–68). ALM, appendicular lean mass.

**Table 2:** Associations of body fat mass percentage and ALM (independent variables) with pain and patient‐reported physical function scales (dependent variables) represented as regression coefficients (95% confidence intervals)*

	Total		Female patients		Male patients	
	Unadjusted (n = 160)	Adjusted (n = 157)^a^	Unadjusted (n = 104)	Adjusted (n = 102)^a^	Unadjusted (n = 56)	Adjusted (n = 55)^a^
Pain						
Body fat mass percentage	0.01 (−0.02 to 0.04)	0.00 (−0.03 to 0.03)	0.03 (−0.02 to 0.08)	0.02 (−0.03 to 0.07)	−0.01 (−0.08 to 0.07)	−0.03 (−0.12 to 0.06)
ALM	0.01 (−0.04 to 0.05)	0.01 (−0.03 to 0.06)	0.04 (−0.07 to 0.15)	0.00 (−0.11 to 0.12)	0.05 (−0.06 to 0.16)	0.02 (−0.10 to 0.15)
Physical function						
Body fat mass percentage	−0.06 (−0.27 to 0.14)	−0.05 (−0.27 to 0.17)	0.24 (−0.10 to 0.59)	0.25 (−0.12 to 0.62)	−0.08 (−0.60 to 0.46)	−0.20 (−0.79 to 0.40)
ALM	0.25 (−0.06 to 0.55)	0.20 (−0.13 to 0.53)	−0.11 (−0.83 to 0.61)	−0.26 (−1.05 to 0.52)	0.27 (−0.51 to 1.05)	0.03 (−0.80 to 0.86)

* ALM, appendicular lean mass.

^a^ Adjusted for age, study site, symptom duration, and Kellgren‐Lawrence grade.

Among the four body composition groups (high body fat, n = 86; low lean mass, n = 11; combined high body fat and low lean mass, n = 8; and neither characteristic, n = 55), there were no significant differences in mean pain or patient‐reported physical function scores. These results are presented in Figure 2, with group‐specific scores, comparative differences, and 95% CIs reported in Table 3.

**Figure 2:**
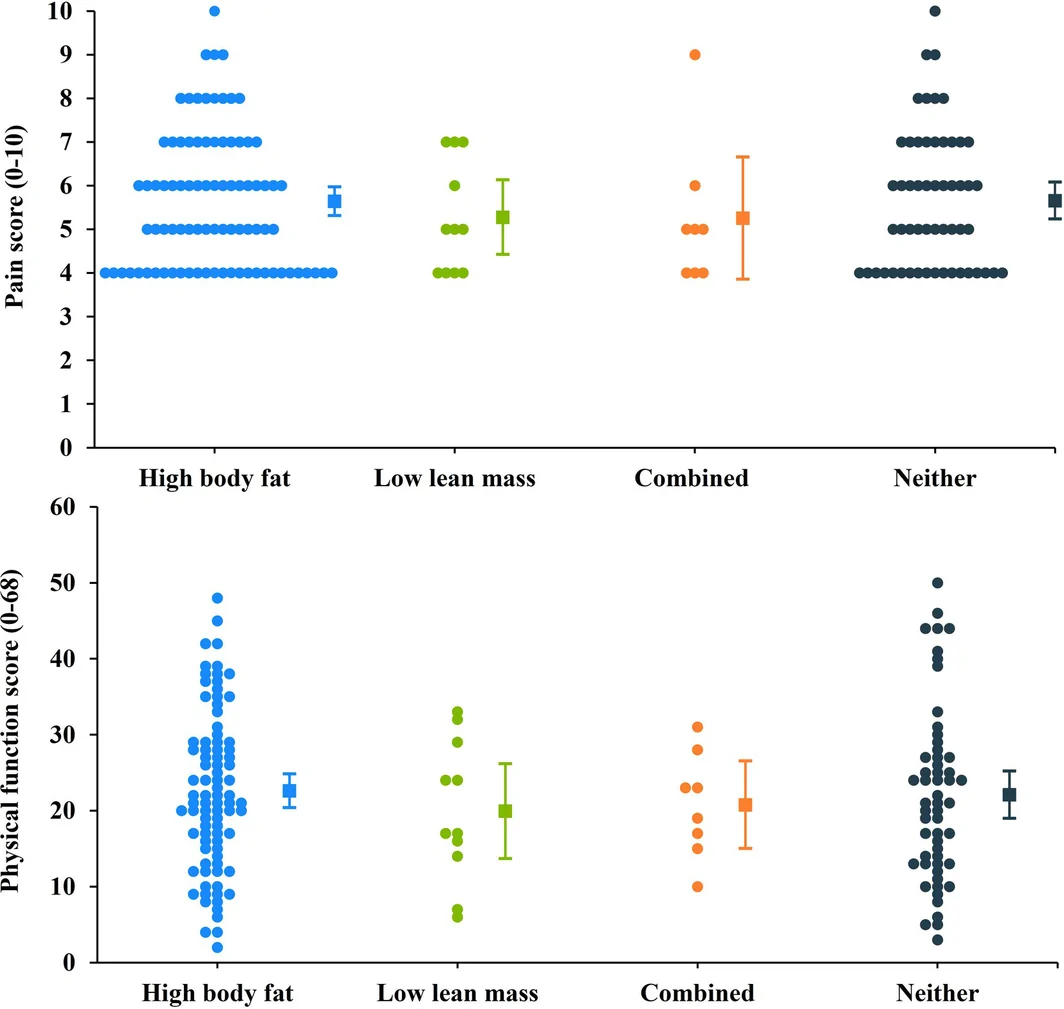
Pain (Numeric Rating Scale, 0–10) and patient‐reported physical function (Western Ontario and McMaster Universities Osteoarthritis Index, 0–68) scores across body composition profiles. The four groups are high body fat alone (n = 86), low lean mass alone (n = 11), combined (high body fat and low lean mass; n = 8), and neither (n = 55). Individual data points and group mean values with 95% confidence intervals are shown.

**Table 3:** Pain and patient‐reported physical function outcomes across body composition phenotypes, with mean differences (95% CI) calculated as reference group minus comparison group, where the reference group is either the combined phenotype or the neither phenotype*

					Combined group, mean difference (95% CI)			Neither group, mean difference (95% CI)		
	High body fat (n = 86), mean (SD)	Low lean mass (n = 11), mean (SD)	Combined (n = 8), mean (SD)	Neither (n = 55), mean (SD)	High body fat	Low lean mass	Neither	High body fat	Low lean mass	Combined
Pain outcomes										
NRS (0–10)	5.6 (1.6)	5.3 (1.3)	5.3 (1.7)	5.7 (1.6)	−0.4 (−1.2 to 0.7)	−0.02 (−1.4 to 1.4)	−0.4 (−1.6 to 0.8)	0.02 (−0.5 to 0.5)	0.4 (−0.6 to 1.4)	0.4 (−0.8 to 1.6)
Physical function outcomes										
WOMAC (0–68)	22.6 (10.4)	19.9 (9.3)	20.8 (6.9)	22.1 (11.5)	−1.8 (−9.6 to 5.9)	0.8 (−8.9 to 10.6)	−1.3 (−9.3 to 6.6)	−0.5 (−4.1 to 3.1)	2.2 (−4.7 to 9.1)	1.3 (−6.6 to 9.3)

* CI, confidence interval; NRS, Numeric Rating Scale; WOMAC, Western Ontario and McMaster Universities Osteoarthritis Index.

## DISCUSSION

Our cross‐sectional analysis found that neither body fat mass percentage nor ALM was associated with pain severity or patient‐reported physical function in individuals with hip OA. Similarly, pain and patient‐reported physical function did not differ across body composition profiles defined by high body fat, low lean mass, their combination, or neither. Collectively, these results suggest that DEXA‐based body composition measures may have limited the ability to distinguish symptom severity in hip OA.

To the best of our knowledge, this is the first study to examine body composition in relation to symptom burden in patients with clinically diagnosed hip OA. Our association analysis findings differ from recent studies in knee OA that have reported associations between higher fat mass and greater knee pain and worse patient‐reported physical function,^5^ or between greater lean body mass and better patient‐reported physical function,^6^ using bioelectrical impedance analysis (BIA). However, it should be noted that differences in methodology (DEXA vs BIA) and in the specific measures used (body fat mass percentage vs absolute body fat mass, and patient‐reported physical function vs performance‐based measures of physical function) could limit direct comparability. In addition to these methodologic differences, hip and knee OA differ in their underlying biomechanics, clinical presentation, and pain mechanisms.^31^ For example, the knee joint has a smaller contact area between articulating surfaces and therefore experiences greater point loading,^32^ whereas the hip's ball‐and‐socket structure provides greater conformity and distributes forces across a larger surface,^33^ which may make adiposity‐related loading more influential in knee OA symptoms. Differences in joint metabolic environments may also contribute to joint‐specific symptom patterns and warrant further investigation.^34^ Furthermore, knee OA has been more strongly linked to muscular and neuromuscular stabilization demands,^31^ whereas hip OA is often characterized by restricted range of motion and movement adaptations,^35^ which may not be adequately captured by measures of muscle quantity such as ALM. As there is increasing recognition that hip and knee OA may differ in their underlying mechanisms and treatment responses, future research using comparable methods and outcomes across joint sites may help clarify these differences.

The study hypothesis that higher body fat mass percentage would be associated with greater pain severity and worse patient‐reported physical function in people with hip OA was not supported. Although greater adiposity may theoretically increase joint load and systemic inflammation, evidence from cohort studies is inconsistent regarding whether weight loss improves hip symptoms^36^, ^37^ or influences the likelihood of hip replacement.^38^ Findings from our recent diet‐induced weight loss intervention reflect this uncertainty. Participants lost approximately 9% of body weight when combining diet with exercise, but this did not lead to consistently greater improvements in hip pain at 6 months compared with exercise alone, although patient‐reported physical function did improve.^14^ This may reflect the complex and variable nature of pain in OA,^39^ which might require more individualized management strategies that target specific pain mechanisms (eg, central sensitization vs peripheral nociception) rather than a generic body composition metric. The improvement in patient‐reported physical function observed during the weight loss intervention, without a corresponding association in our baseline cross‐sectional data, suggests that the benefits of a structured program may be partly mediated by factors other than changes in body composition alone.

Previous cross‐sectional analysis has revealed that hip muscle strength is associated with pain and physical function,^40^ and resistance training produces small‐to‐moderate improvements in hip OA symptoms.^41^ In the current study, however, ALM, a contributor to muscle strength,^42^ was not associated with symptom severity. Additional sensitivity analyses using lower‐ and upper‐limb lean mass separately yielded similar findings, suggesting that the absence of associations was unlikely to be explained by the use of total ALM. This suggests that muscle quantity alone may not be the primary muscular factor linked to symptoms in hip OA. This distinction aligns with the broader understanding of sarcopenia,^20^ in which muscle weakness is recognized as a composite of deficits in mass, strength, and functional capacity, meaning ALM alone provides an incomplete indication of impairment. Therefore, our results indirectly suggest that, for individuals with hip OA, muscle strength and physical performance may be more relevant to symptom severity than lean mass alone.

The impact of sarcopenia and sarcopenic obesity has garnered increasing interest in recent years, although most studies have not considered muscle strength or physical function in their definitions.^26^ Furthermore, definitions of obesity vary widely across studies.^23^, ^24^ To investigate hip OA specifically and explore symptom burden across body composition profiles, we adopted a classification approach from knee OA research based on lean mass and body fat, without overemphasizing conditions such as sarcopenia or sarcopenic obesity. Consistent with the association analyses, our findings indicate that no differences in pain or patient‐reported physical function were observed across the four body composition categories in hip OA. This contrasts with knee OA studies suggesting that the combination of high body fat and low lean mass may exacerbate symptoms.^23^, ^43^, ^44^ For instance, Manoy and colleagues^44^ found that individuals with end‐stage knee OA who had both higher BMI (≥25 kg/m^2^) and lower lean mass (ALM/body mass × 100, with cutoffs of <30.44% for men and <25.8% for women) had higher pain and disability compared with those with neither characteristic. Similarly, Kim et al^43^ reported that knee pain was more prevalent among individuals with the combined phenotype (ALM/body mass ≥2 SDs below sex‐matched young reference values and BMI ≥25 kg/m^2^) compared with those who had neither characteristic nor low lean mass alone. However, variation in the definitions of high body fat and low lean mass across studies may contribute to uncertainty.^23^ For example, BMI‐based thresholds cannot distinguish between fat and muscle,^24^, ^45^ meaning that individuals with high muscle mass may be misclassified as having high adiposity.

Taken together, both the association and categorization results suggest that body composition alone may not adequately capture factors associated with symptom severity in hip OA. However, these findings should be interpreted in the context of the study cohort. Several methodologic considerations may also be relevant. First, DEXA‐derived ALM represents lean soft tissue (eg, muscle, water, and connective tissue) rather than true muscle mass,^46^ which may partly explain the absence of observed associations. Previous studies using DEXA have often identified weak or inconsistent associations, such as those between lean mass and the risk of falls, that become apparent when more precise measures of muscle mass are used.^47^ More precise measures of muscle mass, such as D3‐creatine dilution,^46^, ^47^ may provide greater insight into the muscular factors associated with hip OA symptoms. Second, physical function in the present study was assessed using the WOMAC physical function subscale, a patient‐reported outcome that reflects perceived difficulty performing activities rather than objective physical capability.^29^ Self‐reported and performance‐based functional measures capture related but distinct constructs and are often only moderately correlated in individuals with OA.^48^, ^49^ Therefore, relationships between body composition and functional capacity may differ when objective performance‐based functional tests are used.

Additionally, few participants had low lean mass alone (6.9%) or combined low lean mass and high body fat (5%), which may have limited the statistical power of the categorical analyses. However, this likely reflects the true distribution of these phenotypes in our community‐based hip OA cohort.^24^ Applying full diagnostic criteria for sarcopenia or sarcopenic obesity, which incorporate muscle strength and physical performance, would likely further reduce the number of individuals meeting these definitions,^26^ highlighting the practical challenges of studying these phenotypes in hip OA populations. Simpler classification methods, such as grouping participants by low versus high muscle strength may therefore be more feasible and warrant future investigation. Finally, different DEXA devices were used across sites, and, although manufacturer instructions were strictly followed, cross‐validation was not performed. Previous studies comparing the Lunar and Hologic DEXA systems have reported high correlations for body composition measures, although systematic differences between devices may exist.^50^

In conclusion, in this cohort of patients with clinically diagnosed hip OA, body composition measures were not associated with pain severity or patient‐reported physical function, and symptom burdens did not differ across body composition profiles.

## AUTHOR CONTRIBUTIONS

All authors contributed to at least one of the following manuscript preparation roles: conceptualization AND/OR methodology, software, investigation, formal analysis, data curation, visualization, and validation AND drafting or reviewing/editing the final draft. As corresponding author, Dr Wang confirms that all authors have provided the final approval of the version to be published and takes responsibility for the affirmations regarding article submission (eg, not under consideration by another journal), the integrity of the data presented, and the statements regarding compliance with institutional review board/Declaration of Helsinki requirements.

## Supporting information


**Disclosure Form**:

**Supplementary Table 1.** Associations of upper‐ and lower‐limb lean mass (independent variables) with pain and patient‐reported physical function scales (dependent variables), represented as regression coefficients (95% CI).
