# Supplementary material for: Pain and Patient‐Reported Physical Function Did Not Differ Among Body Composition Profiles in Patients With Hip Osteoarthritis
Source: Arthritis Care Res (Hoboken). 2026 May 5;78(11):1732–40. doi: 10.1002/acr.80058 (PMC13710782; doi:10.1002/acr.80058)
Supplement: Supplementary file 2 [file ACR-78-1732-s001.docx]

| **Supplementary Document**  **Supplementary Table 1.** Associations of upper- and lower-limb lean mass (independent variables) with pain and patient-reported physical function scales (dependent variables), represented as regression coefficients (95% CI). | | | | | | | | |
| --- | --- | --- | --- | --- | --- | --- | --- | --- |
|  | **Total** | |  | **Female subjects** | |  | **Male subjects** | |
|  | Unadjusted  (n=160) | Adjusted*  (n=157) |  | Unadjusted  (n=104) | Adjusted*  (n=102) |  | Unadjusted  (n=56) | Adjusted*  (n=55) |
| **Pain** |  | |  |  | |  |  | |
| Upper-limb lean mass | -0.01  (-0.14 to 0.13) | -0.02  (-0.15 to 0.11) |  | 0.03  (-0.13 to 0.21) | 0.02  (-0.15 to 0.20) |  | -0.06  (-0.27 to 0.15) | -0.06  (-0.28 to 0.17) |
| Lower-limb lean mass | -0.02  (-0.08 to 0.03) | -0.02  (-0.08 to 0.05) |  | 0.00  (-0.09 to 0.08) | 0.00  (-0.09 to 0.09) |  | -0.04  (-0.15 to 0.08) | -0.03  (-0.15 to 0.09) |
| **Physical function** |  | |  |  | |  |  | |
| Upper-limb lean mass | 0.33  (-0.57 to 1.22) | 0.28  (-0.64 to 1.20) |  | 0.36  (-0.78 to 1.49) | 0.33  (-0.89 to 1.56) |  | 0.26  (-1.24 to 1.76) | 0.43  (-1.07 to 1.93) |
| Lower-limb lean mass | 0.07  (-0.39 to 0.52) | 0.05  (-0.42 to 0.52) |  | 0.03  (-0.52 to 0.60) | -0.01  (-0.61 to 0.64) |  | 0.14  (-0.64 to 0.93) | 0.29  (-0.50 to 1.08) |
| * Adjusted for age, study site, symptom duration, and Kellgren-Lawrence grade  CI, confidence interval. | | | | | | | | |
